# Communications between Mitochondria and Endoplasmic Reticulum in the Regulation of Metabolic Homeostasis

**DOI:** 10.3390/cells10092195

**Published:** 2021-08-25

**Authors:** Pengcheng Zhang, Daniels Konja, Yiwei Zhang, Yu Wang

**Affiliations:** The State Key Laboratory of Pharmaceutical Biotechnology, Department of Pharmacology and Pharmacy, The University of Hong Kong, Hong Kong SAR, China; u3005061@connect.hku.hk (P.Z.); kdaniels-1@outlook.com (D.K.); u3005377@connect.hku.hk (Y.Z.)

**Keywords:** mitochondria, endoplasmic reticulum, MAM, energy metabolism

## Abstract

Mitochondria associated membranes (MAM), which are the contact sites between endoplasmic reticulum (ER) and mitochondria, have emerged as an important hub for signaling molecules to integrate the cellular and organelle homeostasis, thus facilitating the adaptation of energy metabolism to nutrient status. This review explores the dynamic structural and functional features of the MAM and summarizes the various abnormalities leading to the impaired insulin sensitivity and metabolic diseases.

## 1. Introduction

In mammalian cells, the mitochondrion is the organelle specialized for energy production through the processes of oxidative phosphorylation, tricarboxylic acid (TCA) cycle and fatty acid β-oxidation [1]. Approximately 90% of cellular reactive oxygen species (ROS) are produced from mitochondria during the reactions of oxidative phosphorylation (OXPHOS) via the electron transport chain (ETC) [2]. As an essential powerhouse of mammalian cells, the mitochondrion is crucial for functional regulation of cellular processes, including metabolism, proliferation, survival and death [3,4,5]. Mitochondrial integrity is maintained through a series of quality control processes such as the mitochondrial unfolded protein response (UPR^mt^), antioxidant defense, fusion and fission, biogenesis and mitophagy-mediated removal [6,7]. The function of mitochondria decreases with advancing age, and dysfunctional mitochondria are implicated in the development and progression of metabolic diseases. However, accumulating evidence shows that alterations in mitochondria may evoke defense mechanisms to elicit coordinated protective effects on metabolic health and life span [8,9].

Endoplasmic reticulum (ER) is a membrane-bound organelle that controls the synthesis, folding, maturation and intracellular transport of proteins. ER also plays an essential role in regulating intracellular calcium homeostasis and lipid metabolism [10,11]. Increased demand for protein synthesis and aggregation of misfolded proteins are the main triggers for ER stress and unfolded protein response (UPR^ER^), a conserved transcriptional and translational program to stimulate chaperone production and restore proteostasis [12,13]. During UPR^ER^, the sensor molecules anchored at the ER membrane, including inositol-requiring enzyme 1 (IRE1), activating transcription factor 6 (ATF6) and RNA-dependent protein kinase (PKR)-like ER kinase (PERK), are activated to transduce signals for the induction of various cellular responses [14]. The defective proteins are transported to the cytoplasm for degradation. This process is referred to as ER-associated degradation (ERAD) [15]. In mammals, ER stress represents a defense mechanism that helps the cells to adapt and survive. However, prolonged ER stress increases the risk of metabolic diseases such as obesity, insulin resistance, diabetes and fatty liver injuries [16,17,18,19].

The function and stress signaling of mitochondria and ER are dynamically intertwined, especially in the context of metabolic regulation [20]. ER stress leads to altered mitochondrial structure and function, as well as impaired glucose/lipid homeostasis [21,22]. Disruption in the normal function of mitochondria results in ER stress activation, which induces aberrant insulin signaling [23,24]. As the powerhouse and the building factory of the cells, mitochondria and ER are not only functionally but also structurally linked [20]. The physical contacts between the membranes of the ER and mitochondria are called mitochondria-associated membranes (MAM), ER-mitochondria encounter structure (ERMES) or ER membrane protein complex (EMC) [25,26]. MAM allow the exchange of different ions and metabolites, and play important roles in regulating mitochondrial dynamics/bioenergetics, calcium homeostasis, lipid metabolism, mito/autophagy and cellular apoptosis [20,27]. MAM mark the sites for mitochondrial DNA (mtDNA) synthesis and dynamic regulation [28,29,30]. ER-mitochondria contacts coordinate mtDNA replication with downstream mitochondrial division to distribute newly replicated mtDNA to the daughter mitochondria [30]. Targeting mitochondria and ER crosstalk represents a promising therapeutic approach for numerous diseases, including metabolic and cardiovascular disorders, neurodegeneration and cancer, as well as the aging process. The present review focuses on the structure and function of MAM and its role in mediating the molecular crosstalk between mitochondria and ER under healthy and abnormal metabolic conditions.

## 2. Structure of MAM

**Figure 1:**
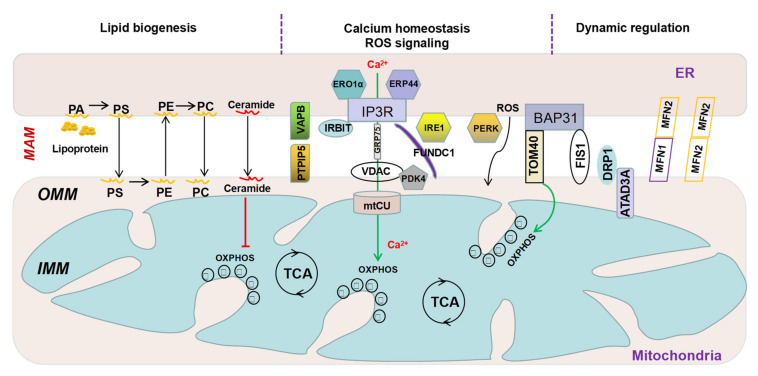
Schematic illustration of the tethering complexes at MAM.

The physical interaction between ER and mitochondria was first proposed over 60 years ago [31,32]. However, such observations were considered artifacts of fixation for a long time. In the 1990s, the existence of MAM was confirmed by biochemical isolation, immunofluorescence labeling and immunoelectron microscopy [33,34,35,36]. The interface of MAM is formed by bringing the two phospholipid bilayers of ER and mitochondria into close proximity (Figure 1). The approximate distance from the outer membrane of mitochondria (OMM) to the ER was originally estimated to be ~100 nm [37]. Subsequent studies using electron tomography reveal a much shorter distance of ~10–50 and ~50–80 nm, respectively, between OMM and the smooth or rough ER [38,39]. The extent of interactions at the juxtaposition between the two organelles appears to be an important parameter of MAM function [40,41]. The formation of MAM relies on proteins expressed on membranes of both ER and mitochondria, which interact either directly or indirectly by forming multiprotein-tethering complexes [42]. Mass spectrometry-based proteomic studies have identified over 1000 proteins participating in the structural and functional interactions at either human or animal MAM samples [43,44]. For example, the ATPase family AAA domain-containing protein 3 (ATAD3) is a single-protein linker for MAM [45]. The vesicle-associated membrane protein B (VAPB), an ER-resident protein, interacts with the tyrosine phosphatase-interacting protein-51 (PTPIP51), an OMM protein to form a complex at MAM [46,47,48]. The ER resident inositol triphosphate receptors (IP3R) physically interact with the cytosolic fraction of the mitochondrial chaperone 75 kDa glucose-regulated protein (GRP75) [49], and the voltage-dependent anion channel (VDAC), a porin ion channel allowing calcium flow through the OMM [50]. The IP3R-GRP75-VDAC tethering complex mediates release of calcium from the ER and the uptake of calcium by mitochondria to stimulate oxidative metabolism [51] (Figure 1).

Proteins that participate in the fission and fusion of mitochondria, including dynamin-related protein 1 (DRP1) in mammals, dynamin 1 (DNM1) in yeast, mitofusion (MFN) 1 and MFN2, are part of MAM [52,53] (Figure 1). MFN2 at MAM forms homo- and heterodimers with mitochondria-localized MFN2 and MFN1, respectively [54]. Deletion of MFN2 reduces mitochondrial calcium uptake induced by inositol-1,4,5-triphosphate (IP3) and causes significant changes in the ER morphology [54]. Ubiquitination of MFN2 by mitochondrial ubiquitin ligase (MITOL) downregulates ER-mitochondria interactions [55]. Phospho-ubiquitination of MFN2 by PTEN-induced kinase 1 (PINK1) and Parkinson protein 2 E3 ubiquitin protein ligase (PARKIN) also triggers the disassembly of ER-mitochondria tethering complexes to drive mitophagy [56]. The PINK1/MFN2/PARKIN pathway is required by the healthy mitochondria to communicate with ER [57]. The presence of MFN2 at MAM facilitates other molecules such as presenilin 2-mediated ER-mitochondrial interactions [58]. Fission and fusion events regulate the structure, distribution and function of mitochondria. ER tubules at MAM are responsible for the constriction of membrane for mitochondrial division [28]. The mitochondrial fission 1 (FIS1), an OMM protein, recruits DRP1 to mitochondrial fission sites [59]. When FIS1 interacts with the B cell receptor-associated protein 31 (BAP31), an ER chaperone located at MAM, apoptosis is induced [60]. BAP31 also interacts with other mitochondrial proteins, including translocase of outer mitochondrial membrane 40 (Tom40), to stimulate the translocation of NADH dehydrogenase (ubiquinone) iron-sulfur protein 4 (NDUFS4), a component of ETC complex I. Disruption of the BAP31-Tom40 tethering complex inhibits mitochondrial ETC complex I activity and oxygen consumption [61].

## 3. MAM and Lipids Biogenesis

MAM possess the ability to synthesize and facilitate the interorganellar exchange of phospholipids and sterols [62,63]. MAM support a robust incorporation of 3H-serine and subsequent conversion into 3H-phosphatidylserine (PS) and 3H-phosphatidylethanolamine (PE) [34]. Various phospholipids, triglycerides, cholesterols and cholesterol esters are present at MAM, which are enriched with enzymes such as diacylglycerol acyltransferase, acyl-coenzyme A, cholesterol acyltransferase, phosphatidylserine synthase, phosphatidylethanolamine N-methyltransferase, PE methyltransferase, PS synthase and decarboxylase [64]. The reactions mediated by enzymes at MAM play a role in enhancing the synthetic efficiency and limiting the dis-semination of the lipid products. For example, the phospholipids generated at MAM are used for lipoprotein assembly in cells. Transport of PS from ER to mitochondria via MAM facilitates its conversion to PE [65,66]. MFN2 binds PS and promotes its transfer to mitochondria for PE biosynthesis [55,56,67]. In yeast, the ERMES complex acts as a transferase to mediate the non-vesicular transport of lipids [68]. In mammalian cells, the PDZ domain-containing protein 8 (PDZD8) is predicted to be structurally similar to the synaptotagmin-like mitochondrial-lipid-binding (SMP) domain proteins [69], and participated in a three-way contact between ER-late endosome and mitochondria [70]. However, it is still unclear if this protein can transport lipids. 

MAM is also considered as a pre-Golgi compartment for the assembly and secretion of lipoproteins [36]. In liver, for instance, MAM contains the microsomal triacylglycerol transfer protein (MTTP), which is required for the biogenesis of lipoproteins that contain apolipoprotein (Apo) B [67]. Nascent ApoB-containing lipoproteins obtained from the lumen of MAM show the same average density and composition as those isolated from heavy and light ER fractions, Golgi, and newly secreted by the cultured hepatocytes [36]. MAM fractions isolated from liver contain mRNA transcripts that encode various proteins for the assembly of plasma lipoprotein particles, such as MTTP, protein disulfide isomerase family A, member 1 (Pdia1), carboxylesterase 1d (Ces1d), ApoB and ApoE [71]. The presence of key components of very low density lipoprotein (VLDL) biogenesis at MAM supports its involvement in lipoprotein bio-genesis, which in turn contributes to systemic lipid metabolism and homeostasis [71].

**Figure 2:**
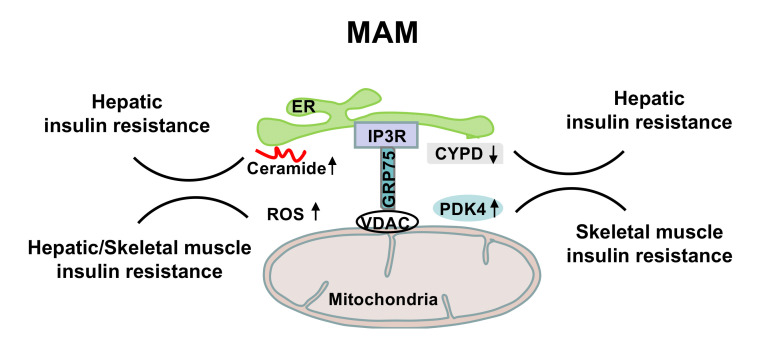
Regulation of MAM on insulin sensitivity.

Sphingolipids, including ceramide, sphingosine, ceramide-1-phosphate (C1P) and sphingosine-1-phosphate (S1P), modulate mitochondrial function and in turn adversely affect cellular redox and energy metabolism [72,73,74]. ER is the primary site for de novo sphingolipid biosynthesis in mammalian cells [75]. Ceramide is translocated through MAM to mitochondria [76,77]. Enzymes for sphingolipid biosynthesis, such as ceramide synthase, ceramidase, sphingomyelinase and sphingosine kinase, have also been detected in the mitochondria and MAM [76,78]. Various ceramide species with different acyl chain attachments are present in the membranes of mitochondria. However, ceramide accumulation inhibits mitochondrial respiration [79,80]. Ceramide transport to the mitochondria by ceramide transfer protein (CERT) induces apoptotic cell death [81]. The medium-chain ceramides form channels in the OMM, which in-crease permeability and enhance release of pro-apoptotic intermembrane space pro-teins, including cytochrome C [82]. Direct interaction between ceramides and VDAC permits cytochrome C release, decreases mitochondrial membrane potential and dis-rupts ETC [83]. Moreover, ceramides inhibit insulin-stimulated protein kinase B (PKB) phosphorylation by activating protein phosphatase A2 and increasing the MAM content of the atypical protein kinase Cζ (PKCζ) [84,85,86,87,88]. Chronic inhibition of ceramide synthesis by myriocin alleviates whole-body adiposity, decreases hepatic inflammation and steatosis, and improves indices of insulin sensitivity [89,90,91]. As one of the main sites for the biosynthesis of sphingolipids, an increased amount of ceramide at MAM negatively affects mitochondrial function and dynamic regulation, thus contributing to the development of insulin resistance and metabolic diseases (Figure 2).

## 4. MAM and Interorganellar Calcium Flux

Mitochondria do not store calcium under physiological conditions. Multiprotein tethering complexes exist at ER-mitochondria junctions to ensure calcium homeostasis for signal coupling [92]. The mitochondrial calcium uniporter (mtCU) located at the inner mitochondrial membrane (IMM) requires calcium levels (10–20 μM) above the cytoplasmic peaks, which can be attained at nanodomains of MAM where IP3R or ryanodine receptors (RyR) calcium release channels (CRC) are located [93]. IP3R and RyR project from the surface of the ER membrane by 10–12 nm, thus limiting the minimum gap distance of MAM [94,95]. The close proximity (≤100 nm) of CRC, mtCU and VDAC allows the local exposure of high calcium flows [49]. However, engineered short linker tighter ER-mitochondria contacts (5 nm) lead to smaller local calcium rise upon IP3R activation than the loose ER-mitochondria contacts via engineered long linkers (15 nm) [41]. GRP75 is critical for transferring calcium to mitochondria through the IP3R CRC [96]. GRP75 silencing attenuates calcium uptake by mitochon-dria by disrupting the functional coupling between IP3R and VDAC [49,97]. The IP3R-GRP75-VDAC complex also interacts with and is regulated by other proteins at MAM. The ER stress transducer IRE1 interacts with the IP3R-GRP75-VDAC complex to stimulate mitochondrial respiration and ATP production [98]. The protein deglycase DJ-1 interacts with IP3R3-GRP75-VDAC to regulate MAM integrity and mitochondrial function [99]. The IP3R binding protein released with IP3 (IRBIT), a molecule that in-teracts with the IP3-binding pocket of IP3R, promotes MAM formation and facilitates calcium transfer to mitochondria [100]. A highly conserved OMM protein, FUN14 domain containing 1 (FUNDC1), interacts with IP3R to modulate the release of calci-um from ER into mitochondria. Disruption of the FUNDC1-IP3R interactions reduces calcium levels in mitochondria [101]. Alterations in the calcium flux at MAM lead to impaired insulin signaling (Figure 2). For example, cyclophilin D (CYPD), a mitochon-drial matrix protein that controls the opening of the permeability transition pore under stress conditions, interacts with the IP3R-GRP75-VDAC complex and facilitates interorganellar calcium exchange at MAM [102,103]. Disruption of MAM integrity by genetic or pharmacological inhibition of CYPD inhibits hepatic insulin signaling. Pyruvate dehydrogenase kinases 4 (PDK4), a mitochondrial enzyme suppressing the conversion of pyruvate to acetyl-CoA, interacts with the IP3R-GRP75-VDAC complex to cause mitochondrial calcium overload and dysfunction, thus dampening skeletal muscle insulin signaling [104].

Ryanodine receptors (RyRs, or feet) are located on the surface of the junctional sarcoplasmic reticulum (jSR) cisterna as two rows of ordered arrays. The RyRs face away from the mitochondrion and the minimum distance between them is 130 ± 45 nm [105]. RyR localizes at MAM in the cardiac muscle [106]. In H9c2 cells, RyR increases calcium levels in mitochondrial matrix, leading to augmented NAD(P)H and ATP production [107,108]. RyR-mediated mitochondrial transfer of calcium via VDAC reg-ulates oxidative metabolism in skeletal and cardiac muscles [109]. Triadin is a key component of the RyR CRC to control the calcium flow form ER [110,111]. In muscle cells, triadin binds to RyR and calsequestrin in a calcium-dependent manner to regu-late the ion channel complex on the membrane of SR. In addition, triadin is also asso-ciated with the MAM protein IP3R to regulate its function [112,113]. Thus, triadin may be essential for regulating the calcium transportation from ER to mitochondria through IP3R or RyR, indirectly regulating mitochondrial metabolism and energy production. Triadin also exhibits a dominant role in the modification of ER structure [114]. Triadin expression was shown to be associated with ER constriction and subsequent narrow ER tube formation. The OMM protein MFN2 regulates calcium levels by interacting with the sarco/endoplasmic reticulum calcium ATPase pump (SERCA), which actively transports calcium back into the ER [115]. Local SERCA activity at MAM controls the background calcium and filters out slow calcium release signals [116]. Of note are a number of activities in MAM that regulate the activity of SERCA. Phosphorylation of transmembrane chaperone calnexin (CNX) has been suggested to abolish the activity of SERCA [117]. Thioredoxin-related transmembrane protein 1 (TMX1) has also been suggested to interact with CNX to abolish the activity of SERCA while promoting ER-mitochondria contact formation [118,119]. Glutathione peroxidase (GPX8), an ER membrane protein and redox regulator resident at MAM, decreases the activity of SERCA [120]. The disruptions of calcium flow in or out from ER and mitochondria represent important processes involved in the development and progression of meta-bolic diseases [121].

## 5. MAM and ROS Signaling

In mitochondria, ROS is mainly produced from OXPHOS of ETC, with complexes I and II being the major culprits [122]. Under specific conditions and above certain thresholds, mitochondrial ROS may function as signaling factors or detrimental molecules to cellular processes. ROS can also be generated from the nanodomains and act as signaling messengers at MAM [123]. ROS originated from the cristae of mitochondria are enhanced by spikes in mitochondria calcium and regulated by a positive feedback loop [124] (Figure 1). Mitochondrial calcium aligns with ROS to regulate the opening of mitochondrial permeability transition pore (mPTP). The signaling of both calcium and ROS is determined according to their concentrations and spatial temporal restrictions [125]. A mild increase in ROS generated at MAM is necessary for cell survival under conditions of oxidative stress by decreasing ER-mitochondria calcium transfer [126,127]. However, excessive production of ROS and sustained calcium accumulation in mitochondria cause disruption of the mitochondrial membrane potential (ΔΨ) and opening of the mPTP, followed by controlled cell death [128]. At this stage, it is still not known how a refined production of ROS at MAM, in alliance with calcium, is achieved at the right time, right amount and right place to maintain cellular homeostasis.

Excessive calcium transfer via MAM induces the overproduction of mitochondrial ROS. For instance, augmented MAM formation results in increased transfer of calcium from the ER to mitochondria, and subsequent overproduction of ROS. Suppression of FUNDC1 and subsequent reduction in MAM formation ameliorate mitochondria ROS overproduction [129]. The different calcium channel regulators resident at MAM play key roles in modulating ROS production. For example, endoplasmic reticulum oxidoreductase 1 alpha (ERO1α) and endoplasmic reticulum protein 44 (ERp44) are ER oxidoreductases highly enriched in MAM [130]. ERO1α causes ERp44 dissociation from IP3R by oxidizing the latter, which results in an exacerbated transfer of calcium from the ER to mitochondria, and subsequent ROS overproduction [131,132]. The increased ROS production further promotes ERO1α-dependent calcium signaling at MAM [133,134,135]. The disrupted-in-schizophrenia 1 (DISC1) dysfunction results in ab-errant mitochondria calcium accumulation following ROS overproduction, leading to mitochondrial abnormalities [136]. In skeletal muscles, disruption of RyR function promotes increased mitochondria ROS production [137]. In a murine model of aging, carbonylation and cysteine nitrosylation of RyR result in increased ROS production and impairment for muscle force generation. Rapamycin treatment-mediated RyR destabilization leads to increased mitochondrial matrix calcium levels, reduced mitochondrial membrane potential and enhanced ROS production [137].

## 6. MAM in the Regulation of Insulin Sensitivity and Energy Homeostasis

Many of the proteins at the interface between ER and mitochondria are involved in metabolic regulation and nutritional or hormonal signaling, highlighting the role of MAM in metabolic homeostasis [138]. Miscommunications between mitochondria and ER contribute to metabolic diseases such as insulin resistance and type 2 diabetes (T2D) [104,110,139,140]. While restoring ER-mitochondria tethering rescue the insulin sensi-tivity [141]. The MAM is essential for mitochondrial calcium signaling and ATP production, which is crucial for metabolic regulation in multiple organs [142].

The formation and structure of MAM are dynamically regulated by changes in the nutritional status [143]. The average length of MAM increases twice in the liver during starvation. Limited nutrients are associated with decreased mitochondria size, cristae density and respiratory capacity, but increased amount of MAM [144]. MFN2 is essential for these changes in response to the nutrition status. A proteolytic inactivation of optic atrophy 1 (Opa1), a major regulator of fusion and cristae architecture, accompanies these changes [144]. Thus, mitochondria adapt to nutrient depletion by coupling the molecular machineries that organize cristae architecture and MAM formation, which were previously thought to operate independently of each other [144]. Disruption of MAM by MFN2 depletion impairs starvation-induced autophagy and the formation of autophagosomes [145]. Glucose represents a main nutritional regulator of hepatic MAM integrity and mitochondrial dynamics and functions, through the pentose phosphate-protein phosphatase 2A (PP-PP2A) pathway [143]. PP2A regulates the release of calcium by IP3R at MAM [146]. High glucose levels reduce PP2A presence at MAM during refeeding in liver [97]. Altogether, MAM could act as a sensor to adapt nutritional transition in cell by regulating mitochondrial metabolism.

The integrity of MAM is necessary for glucose and insulin signaling to regulate metabolic adaptations to nutritional cues [97,147]. Indeed, several proteins associated with insulin signaling were found to locate at MAM interface, including the mammalian target of rapamycin complex 2 (mTORC2), PKB, the protein phosphatase 2A (PP2A), and the phosphatase and tensin homolog (PTEN) [148]. Chronic disruption of MAM by hyperglycemia participates in hepatic mitochondrial dysfunction associated with insulin resistance [143]. The MAM integrity was reported to be damaged by feeding in liver, which could be mimicked by augmenting blood glucose concentration [143]. In consistence, the in vitro experiment revealed that increasing glucose level inhibited the ER-mitochondria communication and Ca^2+^ exchange, suggesting a major role of glucose in regulating MAM integrity under status of high energy. At the molecular level, the pentose phosphate (PP)-PP2A pathway was revealed to mediate the regulation of glucose on MAM integrity and subsequent mitochondrial respiration [143].

Hepatocyte insulin sensitivity has been shown to reduce in both genetically and high fat diet-induced obese and diabetic mice with reduced MAM content and function [104,110,139], demonstrating the importance of MAM in hepatocyte insulin signaling. Antidiabetic medications in obese diabetic mice improve glucose tolerance and metabolism, increased MAM content in liver [97], reduced gluconeogenesis, and reduced ER stress. In the palmitate-induced insulin resistance model, palmitate has been demonstrated by several groups to disrupt MAM, induce ER stress, and reduce insulin signaling, which can be attenuated in hepatocytes by the overexpression of MFN2 in particular [104,139,140]. Another study in which ER-mitochondria tethering molecules MFN2 and GRP75 were silenced to disrupt MAM function showed that insulin signaling was impaired [97]. Disruption of the IP3R-GRP75-VDAC complex by CYPD knockdown also induced systemic insulin resistance and increased hepatic gluconeogenesis [97]. MAM content and function, although still a subject of contention has been widely shown to play a central role in hepatocyte insulin sensitivity and glucose homeostasis in obesity.

MAM content is found to be significantly lower in skeletal muscles of diet-induced and genetically obese mice as well as in obese diabetic humans. Tubbs and colleagues reported an early onset of reduction in MAM content following just 1-week of HFD feeding without insulin resistance, which only became evident after 16-weeks of HFD feeding, an indication that reduction of MAM content could contribute to insulin resistance but not the other way round [147]. A study by Tubbs et al. also showed that a significant disruption of the interactions between ER and mitochondria precedes mitochondrial dysfunction and insulin resistance in the myotubes of obese patients with or without T2D compared to healthy lean subjects. They also showed in both mice and human studies that, defective ER-mitochondria coupling is closely linked to impaired muscle insulin sensitivity [147,149].

In pancreatic β-cells, acute glucose treatment stimulates ER-mitochondria interactions and calcium exchange, whereas disruption of MAM alters glucose-stimulated insulin secretion [150]. Chronic high glucose levels result in a reduced calcium store in ER but an enhanced calcium accumulation in mitochondria, accompanied by ER stress and altered mitochondrial respiration, collectively contributing to glucotoxicity-induced β-cell dysfunction [150]. In pancreatic β-cells of patients with type 2 diabetes, the number of IP3R-GRP75-VDAC complex is significantly decreased due to a re-duction in organelle interactions at MAM, thus leading to a defective glucose-stimulated insulin secretion [151]. ER calcium depletion and mitochondrial calcium overload were observed, which respectively led to ER stress and mitochondria dysfunction, and ultimately to impaired insulin secretion.

In brown adipose tissue, inhibition of ERAD results in the formation of dysfunctional megamitochondria, due largely to increased MAM and ER perforation [139]. The sigma non-opioid intracellular receptor 1 (SigmaR1) is upregulated at MAM to mediate mitochondrial hyperfusion by promoting MFN2 oligomerization [139]. In white adipose tissue, CDGSH iron sulfur domain 2 (Cisd2) at MAM modulates mitochondrial calcium uptake. Loss of Cisd2 causes metabolic defects in patients with Wolfram syn-drome by blunting mitochondrial biogenesis, adipogenesis, insulin-stimulated glucose uptake and the secretion of adiponectin [140,152]. In white adipose tissues, adrenergic stimulation activates PDK4 [153] which has recently been implicated in MAM formation [104].

## 7. Conclusions

The importance of the crosstalk between the ER and mitochondria in regulating organelle homeostasis and cell function cannot be over emphasized. The MAM per se promise to be an important hub where both insulin and nutrient signaling are tightly regulated to ensure metabolic homeostasis. However, the precise relationship between the miscommunications of the two organelles and the development of metabolic diseases remains to be elucidated. It will also be interesting to explore this relationship in humans. Future studies should be directed towards identifying agents that can improve the integrity, flexibility and function of MAM, as they may represent a new generation of therapeutics for prevention and treatment of metabolic diseases.
